# Physicochemical, Textural, and Sensory Properties of Cookies Formulated with Canola Oil-Based Oleogels and Mesquite Flour

**DOI:** 10.3390/foods15122077

**Published:** 2026-06-08

**Authors:** Katherine Meirama-Ross, Jose Alberto Gallegos-Infante, Nuria Elizabeth Rocha-Guzmán, Blanca Elizabeth Morales-Contreras, Silvia Marina González-Herrera, Manuel Pensáben-Esquivel, Roselis Carmona-García, Sonia Guadalupe Sayago-Ayerdi, Alicia Paulina Cardenas-Castro

**Affiliations:** 1 Laboratorio Nacional Conahcyt de Apoyo a la Evaluación de Productos Bióticos (LaNAEPBi), Unidad de Servicio, Tecnológico Nacional de México/I.T. de Durango (TecNM/ITD), Blvd. Felipe Pescador 1830 Ote., Durango 34080, Durango, Mexico; 23040248@itdurango.edu.mx (K.M.-R.); nrocha@itdurango.edu.mx (N.E.R.-G.); bmorales@itdurango.edu.mx (B.E.M.-C.); sgonzalez@itdurango.edu.mx (S.M.G.-H.); alicia.cardenas@itdurango.edu.mx (A.P.C.-C.); 2 TecNM/Instituto Tecnológico de Durango, Durango 34080, Durango, Mexico; mpensaben@itdurango.edu.mx; 3 Tecnológico Nacional de México, Instituto Tecnológico de Tuxtepec, San Juan Bautista Tuxtepec 68350, Oaxaca, Mexico; rosel_car@hotmail.com; 4 Tecnológico Nacional de México, Instituto Tecnológico de Tepic, Av. Tecnológico No 2595, Col. Lagos del Country, Tepic 63175, Nayarit, Mexico; sonia.sayago@gmail.com

**Keywords:** cookie reformulation, oleogel, mesquite flour, storage stability

## Abstract

The reformulation of cookies using alternative flours and structured lipid systems represents a promising strategy for improving their nutritional profile. The present study characterized the dough properties, baking behavior, compositional attributes, and 48-day storage physicochemical and textural stability of cookie formulations combining mesquite or wheat flour with varying proportions of shortening and monoglyceride-based oleogel. A multifaceted modeling and temporal analysis approach was employed to assess the impact of flour type, fat blend, and storage duration on critical physicochemical variables. The findings of the study indicated that the type of flour was the predominant factor influencing moisture retention, ash content, and the rate of bake loss. In contrast, the fat blend was found to regulate oil migration and dough mechanical parameters. Oleogel-rich systems demonstrated superior stability over time, as evidenced by a diminished color change and a decelerated textural hardening process in comparison to conventional shortening controls. Concurrently, these systems maintained water activity levels below the established microbiological safety thresholds. Sensory analysis demonstrated that oleogels effectively replicated the mouthfeel and acceptability of conventional fats, exhibiting comparable hardness and crunchiness to traditional formulations. However, mesquite flour-rich formulations exhibited higher bitterness and lower adhesiveness. These findings demonstrate that oleogel incorporation provides a viable strategy for mitigating textural staling and improving lipid profiles of cookies.

## 1. Introduction

Dietary patterns have undergone substantial changes in recent decades, primarily due to a shift toward the consumption of highly processed foods high in saturated fatty acids (SFAs), trans fatty acids (TFAs), added sugars, and sodium. This transition has been associated with the rising global prevalence of non-communicable chronic diseases, including cardiovascular disease, obesity, type 2 diabetes mellitus, dyslipidemia, and certain forms of cancer. The health implications for individuals and public health systems have prompted the implementation of regulations aimed at reducing exposure to nutritionally detrimental lipid fractions, particularly trans fatty acids (TFAs) from partially hydrogenated vegetable oils [1].

In response to the mounting regulatory constraints imposed on the utilization of partially hydrogenated oils in the food manufacturing sector, the food industry has been compelled to identify technologically viable alternatives that can substitute for solid fats while maintaining the sensory and functional characteristics of finished products [2]. Among the most promising developments in this area are oleogels, which are structured lipid systems in which a liquid oil phase is trapped within a three-dimensional supramolecular network created by gelling agents.

In contrast to conventional solid fats, whose functional properties are derived from the crystalline arrangement of saturated or partially hydrogenated fatty acids, oleogels achieve analogous rheological characteristics through the physical trapping of the oil [3,4]. This structural approach enables a substantial reduction in SFA and TFA contents while preserving the plasticity, mouthfeel, and stability essential for baked goods and other fat-rich products [5,6].

Canola oil has garnered significant attention as a constituent of oleogel formulations due to its low saturated fat content, substantial monounsaturated oleic acid, and favorable oxidative stability. The substance’s extensive regulatory approval for food use, as well as its endorsement by the U.S. Food and Drug Administration, serves to further substantiate its suitability for industrial applications [7,8]. The incorporation of gelling agents during the structuring of canola oil enables the production of oleogels that exhibit rheological profiles analogous to those of commercial shortenings. This renders oleogels promising candidates for direct substitution in baked product formulations [9].

The mounting interest in the diversification of staple ingredients has prompted a shift in focus toward non-conventional flours as potential substitutes for refined wheat flour. Among these, mesquite flour derived from the pods of *Neltuma laevigata* has emerged as an ingredient of notable nutritional value. Mesquite flour is characterized by its high dietary fiber content (16.9–41 g/100 g), appreciable protein levels (9.5–24.71%), and a low glycemic index attributable to the presence of galactomannans, resistant starch, elevated concentrations of essential minerals (potassium, calcium, magnesium, iron), and significant amounts of bioactive phenolic compounds [10].

Notwithstanding the global preeminence of the cookie market, the combination of oleogels and mesquite flour has not yet been the subject of investigation. A body of research has demonstrated the efficacy of oleogels in substituting saturated fats in cookies. However, concerns have been raised regarding the potential compromise of structural integrity that this substitution may entail. Concurrently, mesquite flour exhibits underutilization in baking contexts despite its favorable nutritional characteristics. However, its application is hindered by unfavorable effects on dough rheology and flavor profile. By exploring the synergy between these two ingredients, researchers can address a shifting consumer demand for healthier profiles, potentially simultaneously overcoming the individual technical challenges of fat reduction and nutritional enrichment.

The objective of the present study was to evaluate the effect of partial or total substitution of commercial shortening with canola oil-based oleogel, combined with the partial replacement of wheat flour with mesquite flour, on the physicochemical, textural, and sensory properties of cookies. The study’s objective was twofold: first, to ascertain the impact of these formulations on the physicochemical and textural stability of the products over the course of a 48-day storage period, and second, to contribute to the development of healthier alternatives in the biscuit and confectionery market.

## 2. Materials and Methods

### 2.1. Raw Materials

The selection of food-grade pure canola oil (Canoil, AGYDSA, Guadalajara, Jal, México) was made based on its notably low saturated fat content and high monounsaturated fatty acid content. The oil was stored in hermetically sealed containers in a cool, dark environment prior to use to prevent oxidative degradation. The commercial shortening (Hill Country Fare brand, San Antonio, TX, USA) was maintained as a standard commercial reference for comparison of physicochemical properties with the experimental oil phases. The supply of fatty materials was sourced from a certified supplier in Durango, Mexico.

Refined wheat flour (Selecta brand, Manila, Philippines) was obtained from a local certified food-grade supplier and stored in a dry, cool environment to prevent deterioration prior to use. Mesquite flour was obtained from mature *Neltuma laevigata* pods, harvested in July 2020 from the Valle del Guadiana (Instituto Nacional de Investigaciones Forestales, Agrícolas y Pecuarias [INIFAP], Durango, Mexico). Following a meticulous selection process aimed at ensuring the highest standards of quality, the pods were meticulously washed, dried at a temperature of 40–50 °C, and subsequently milled into a fine flour with a diameter of less than 0.6 mm.

### 2.2. Cookie Formulation and Preparation

The development of eight cookie formulations was conducted using a factorial design, incorporating two distinct types of flour and four different fat compositions. The fixed ingredients included sucrose (12.11%), sodium chloride (0.04%), and sodium bicarbonate (0.04%). The flour treatments comprised two distinct groups: 100% wheat flour (T, 60.56%) and a 75:25 wheat-mesquite blend (M, 45.42%:15.14%). The fat phases (27.25% total) comprised shortening (S) and oleogel (O) at ratios of 100:0 (S100), 67:33 (S67O33), 33:67 (S33O67), and 0:100 (O100). The oleogels were prepared by mixing canola oil with 3% *w*/*w* Myverol™ 18-04 PK (Kerry, SW Food Tecnología, Monterrey, Nuevo León, Mexico). The mixture was subsequently heated to 80 °C and stirred at 1000 rpm for 10 min; the purpose of this step was to ensure complete dissolution of the gelator. The resultant mixture was subsequently transferred into glass containers and permitted to undergo gelation over a period of 24 h at a temperature of 13 °C, with a standard deviation of ±2 °C, prior to analysis. The eight resulting treatments were designated as TS100, TO100, TS33O67, TS67O33, MS100, MO100, MS33O67, and MS67O33.

The cookies were meticulously crafted using a KitchenAid Stand Mixer (K45SS) equipped with a flat beater. The fat phase (comprising shortening, oleogel, or a blend) was creamed at medium speed for two minutes, followed by an additional four minutes after the addition of powdered sugar. The dry ingredients (wheat flour, mesquite flour, salt, and baking powder) were then incorporated at a low speed for a period of eight minutes, with the objective of ensuring homogeneity. The dough was subsequently shaped into disks and baked at 135 °C for 20 min on parchment-lined trays. Following the baking process, the cookies were allowed to cool on wire racks at room temperature for a period of 60 min prior to analysis. The formulation and cooking parameters were established in accordance with preliminary optimization trials.

### 2.3. Dough Properties

#### 2.3.1. Textural Properties of Cookie Dough

The textural characteristics of the cookie doughs were evaluated using a TA-XT Plus Texture Analyzer (Stable Micro Systems, Surrey, UK) equipped with a 6 mm cylindrical probe. Samples were meticulously packed into 3 × 3 cm containers to eliminate the presence of air bubbles and were measured at a temperature of 25 °C. Test velocities were set at 5.0, 2.0, and 10 mm per second (mm/s) for the pre-test, test, and post-test phases, respectively. The trigger force was set at 5 g, and the deformation was set at 50%. The recorded parameters included peak positive force (N), positive area (N·s), negative area (N·s), and slope to positive peak (N/s).

#### 2.3.2. Baking Transformation Indicators

The total spread ratio was calculated as the ratio between the spread factor of the baked cookie (width/thickness) and the corresponding spread factor of the unbaked dough, measured with a ruler before and after a 60 min cooling period. The mass loss rate was determined gravimetrically by first weighing the dough before baking and then the cookies after baking. The mass loss rate, expressed as a percentage, was calculated by dividing the difference between the post-baking cookie weight (W1) and the pre-baking dough weight (W2) by 100. The moisture content was ascertained prior to and following the baking process, in accordance with the methodology delineated in Section 2.4.1. The Moisture Retention Index (%), a measure of the extent to which moisture is retained by a material, was calculated as follows: (Hf/Hi) × 100, where Hf denotes the final moisture content of the baked cookie and Hi signifies the initial moisture content of the dough.

### 2.4. Physicochemical Characterization of Cookies

#### 2.4.1. Moisture Content

The moisture content of both dough and baked cookies was determined by oven-drying at 105 °C until constant weight was achieved, as previously described [11].

#### 2.4.2. Ash Content

The ash content of cookies was determined by incineration in a muffle furnace at 550 °C, adhering to standard AOAC criteria [11,12].

#### 2.4.3. Protein Content

Total protein content was quantified through the Kjeldahl method [11,13].

#### 2.4.4. Oil Migration

Each cookie was placed on a pre-weighed absorbent paper under controlled conditions (28 ± 1 °C) for 24 h. The absorbent paper was re-weighed after the contact period, and oil migration (mg oil/cookie) was calculated as the mass difference of the paper before and after the test. All measurements were performed in triplicate.

#### 2.4.5. Triacylglycerol Profiling by UPLC-ESI-MS/MS

A relative triacylglycerol (TAG) profile of cookie samples was obtained by ultra-performance liquid chromatography coupled with electrospray ionization tandem mass spectrometry (UPLC-ESI-MS/MS), targeting 34 TAG species. For the lipid extraction process, approximately 0.5 g of finely ground cookie was extracted with 4 mL of hexane for a period of 6 h. This extraction was carried out using an Ultra-Turrax disperser, with the mixture being stored in darkness to prevent degradation. The analysis was not guided by the implementation of internal standards, as the primary objective was to assess relative profile percentages. The extract was subjected to a centrifugal process at 10,000 rpm for a duration of 5 min at a temperature of 4 °C. The hexane phase was then subjected to evaporation, employing a centrifugal evaporator (Labconco, Kansas City, MO, USA), until it was completely dry. The dry residue was then reconstituted in 200 µL of acetonitrile, filtered through a 13 mm PVDF syringe filter with a 0.45-µm pore size, and dispensed directly into a vial insert with perforated septa. The chromatographic separation was carried out on an Acquity^®^ UPLC CSH C18 column (1.7 µm, 100 mm × 2.1 mm; Waters Corp., Milford, MA, USA) maintained at 60 °C, with an injection volume of 0.10 µL. The mobile phase comprised solvents A and B, namely water and acetonitrile, respectively. The gradient profile initiated with 85% A and 15% B before increasing to 1% A and 99% B, followed by a return to the initial conditions. This process was executed at a constant flow rate of 0.300 milliliters per minute. The detection process was executed through the utilization of a XEVO-TQS mass spectrometer (Waters Corp., Milford, MA, USA) operating in positive electrospray ionization (ES+) mode under the framework of Multiple Reaction Monitoring (MRM). This instrument was configured to scan a mass range of 400–1000 Da, with a collision energy setting of 2.0 eV. The source temperature was set at 150 °C, the desolvation temperature was set at 450 °C, the cone gas flow was set at 150 L/h, the desolvation gas flow was set at 800 L/h, and the capillary voltage was set at 3.50 kV. The identification of individual TAG species was based on their specific MRM precursor/product ion transitions (which correspond to the neutral loss of individual fatty acyl chains). For the purpose of analysis, TAGs were classified into two groups based on their objective fatty acid profiles: UFA-rich TAGs (predominantly composed of monounsaturated and polyunsaturated fatty acids) and SFA-rich TAGs (predominantly composed of saturated fatty acids).

### 2.5. Storage Physicochemical and Textural Stability Study

#### 2.5.1. Experimental Design and Storage Conditions

Following preparation, cookies were vacuum sealed to minimize exposure to at-mospheric oxygen and external moisture and stored under controlled conditions at 24 ± 2 °C and 21 ± 3% relative humidity in calibrated climate chambers, representative of typical domestic storage environments. Evaluations were conducted at three time points: day 0 (baseline), day 24 (midpoint), and day 48 (end of study).

#### 2.5.2. Analyses During Storage

Water activity (a*_w_*) was measured using an AquaLab 4TE water activity meter (METER Group, Inc., Pullman, WA, USA). The measurements were conducted at a temperature of 25 °C and recorded as the mean of three independent replicates, expressed as absolute a*_w_* values. The pH was determined in accordance with the AOAC 943.02 potentiometric method on pulverized and homogenized cookie samples. The color of the pulverized, homogenized cookie samples was evaluated using a colorimeter configured under the CIE L*, a*, b* system. In this system, lightness (L*), redness (a*), and yellowness (b*) values were recorded. The color difference between the two days was calculated using the Δ*E* (Delta *E*) formula. This formula measures the distance between two points in the CIELAB color space:

(1) $$\Delta E=\sqrt{{{(L}_{2}^{*}-L_{1}^{*})}^{2}+{\left(a_{2}^{*}-a_{1}^{*}\right)}^{2}+{\left(b_{2}^{*}-b_{1}^{*}\right)}^{2}}$$

Texture was assessed using the TA-XT Plus texture analyzer (Stable Micro Systems, Surrey, UK), which was equipped with a 20 mm cylindrical probe (P/20) and a high-resistance platform (HDP/90) with a perforated plate. Test velocities were set at 5.0, 1.0, and 10 mm per second (pre-test, test, and post-test, respectively) with a 5 g trigger force and 50% deformation. The recorded parameters included hardness (peak force, N), crunchiness (slope to peak positive, N/s), and adhesiveness (negative peak force and negative area, N·s). These parameters represent the force and energy required for probe detachment. The force exerted at the target was meticulously documented.

### 2.6. Sensory Evaluation Methodology

Sensory analysis was conducted in individual evaluation booths under controlled lighting and temperature conditions. Considering the preceding analysis, the formulations that were selected were as follows: TS100, TS33067, TO100, MS33067, and MO100. A quantitative descriptive analysis (QDA) was conducted to assess the intensity of selected sensory attributes in cookies. The evaluation of the attributes included the bitter taste, the oily/greasy mouthfeel, the adhesiveness/stickiness, the crunchiness, and the hardness. The study’s panel comprised 21 trained assessors within the 18–30 age range. The calibration and orientation of the panelists was conducted over the course of two one-hour sessions. In these sessions, the panelists were aligned using reference standards for each attribute. The attributes of bitterness were aligned using an aqueous solution of caffeine, while the attributes of mechanical texture and lipid-related mouthfeel were aligned using commercial cookie standards. Each panelist was provided with a coded three-digit randomized cookie sample and was instructed to rate each attribute on a nine-point intensity scale ranging from 1 (“not at all”) to 9 (“very intense”). To complement the descriptive data, an internal product screening was conducted with the same 21 panelists to assess preliminary overall acceptability. To prevent the influence of peer interaction on the results, the panelists were instructed to evaluate the product acceptability individually and independently within the isolated evaluation booths using a nine-point liking scale (1 = “dislike extremely”; 9 = “like extremely”). All experimental activities were approved by the relevant Ethics Committee of TecNM/ITD (CEI-003-2022-0301-025). Informed consent was obtained from each participant prior to their inclusion in the study.

### 2.7. Statistical Analysis

A statistical analysis was conducted, which involved the implementation of two principal component analyses (PCAs). The first principal component analysis (PCA) was performed on dough properties and cookie composition, while the second PCA was conducted on storage stability. Subsequently, a two-way analysis of variance (ANOVA) was employed to assess the impact of flour type (Flour Type) and fat composition (Fat Mix), incorporating their interaction. Furthermore, a three-way ANOVA was conducted to assess the combined effects of flour type, fat composition, and storage time (Time), along with all relevant two-way and three-way interactions. Tukey’s HSD test revealed significant differences (*p* < 0.05). The statistical analyses and figures were generated using R (v. 4.5.2) in RStudio (v. 2026.1.1.403).

## 3. Results

### 3.1. Physicochemical Characterization of Dough and Cookie Properties

#### 3.1.1. Multivariate Structure of Dough, Baking, and Compositional Variables

**Figure 1:**
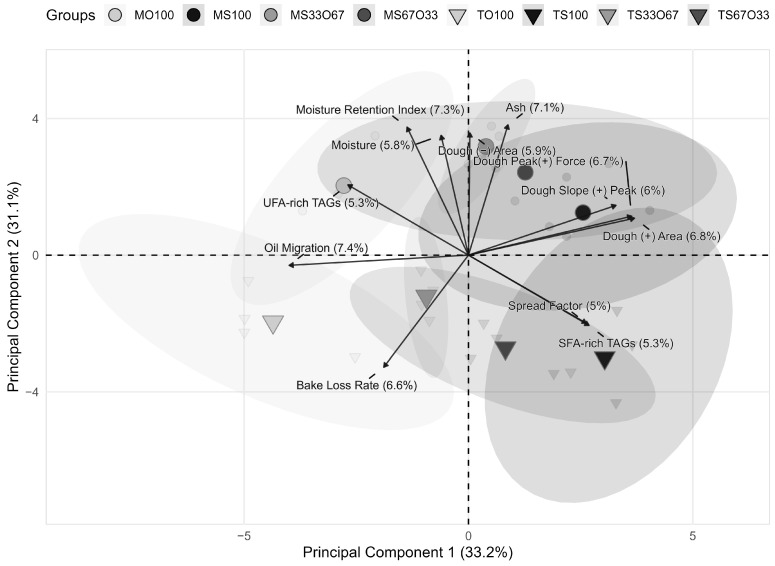
Principal component biplot of mass and product properties in wheat and mesquite-based cookies with shortening and oleogel fats. Vectors indicate variable direction, labeled with their total contribution percentage across both components (PC1 + PC2); ellipses represent 95% confidence intervals for each sample group. T = Wheat-based; M = Mesquite-based; S = Shortening; O = Oleogel.

Principal component analysis performed on the combined dough, baking, and compositional variables of the cookies (PCA1) yielded a two-dimensional solution that explained 64.3% of the total variance (Principal Component 1 (PCA1) = 33.2%; Principal Component 2 (PCA2) = 31.1%) (Figure 1).

**Figure 2:**
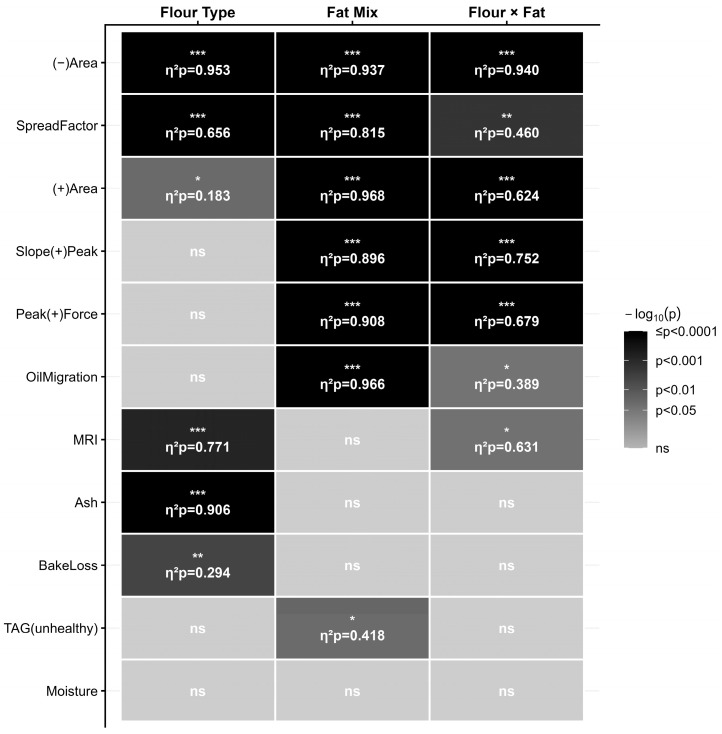
Heatmap of ANOVA effects on quality variables (PCA 1) in wheat and mesquite cookies. Cell values display the statistical significance alongside the practical effect size using partial eta-squared (η^2^p); color intensity represents −log10(*p* value). Stars denote significance levels: *** *p* < 0.001, ** *p* < 0.01, * *p* < 0.05, ns = not significant.

TS and TO formulations clustered in the negative PCA2 region, which was characterized by higher bake loss and lower moisture retention. ANOVA highlighted Flour Type as a highly significant factor (*p* < 0.001), which suggests that PCA2 captures variability strongly associated with flour composition rather than fat structure (Figure 2).

#### 3.1.2. ANOVA of Formulation Factors

To identify the experimental factors linked to the variation captured by the first principal component, a two-way ANOVA was conducted considering Flour Type, Fat Mix, and their interaction (Flour × Fat) for the variables with the highest loadings on PCA1 and PCA2 (Figure 2). Pairwise comparisons (Tukey HSD) for key physicochemical parameters are shown in Table 1.

MS100 and TS100 showed the highest Dough (+) Area values (16.71 and 14.74 N·s), which aligns with the greater stiffness typically observed in shortening-based systems. Despite varying dough consistency, the TS series (TS100, TS33O67, TS67O33) consistently achieved the highest Spread Factors (2.33–2.48). This co-variation indicates that spread in wheat formulations is strongly related to the fat-flour interaction, rather than being modulated by dough firmness alone.

### 3.2. Physicochemical and Textural Stability of Cookies During Storage

#### 3.2.1. Multivariate Structure, Formulation Identity, and Temporal Dynamics

**Figure 3:**
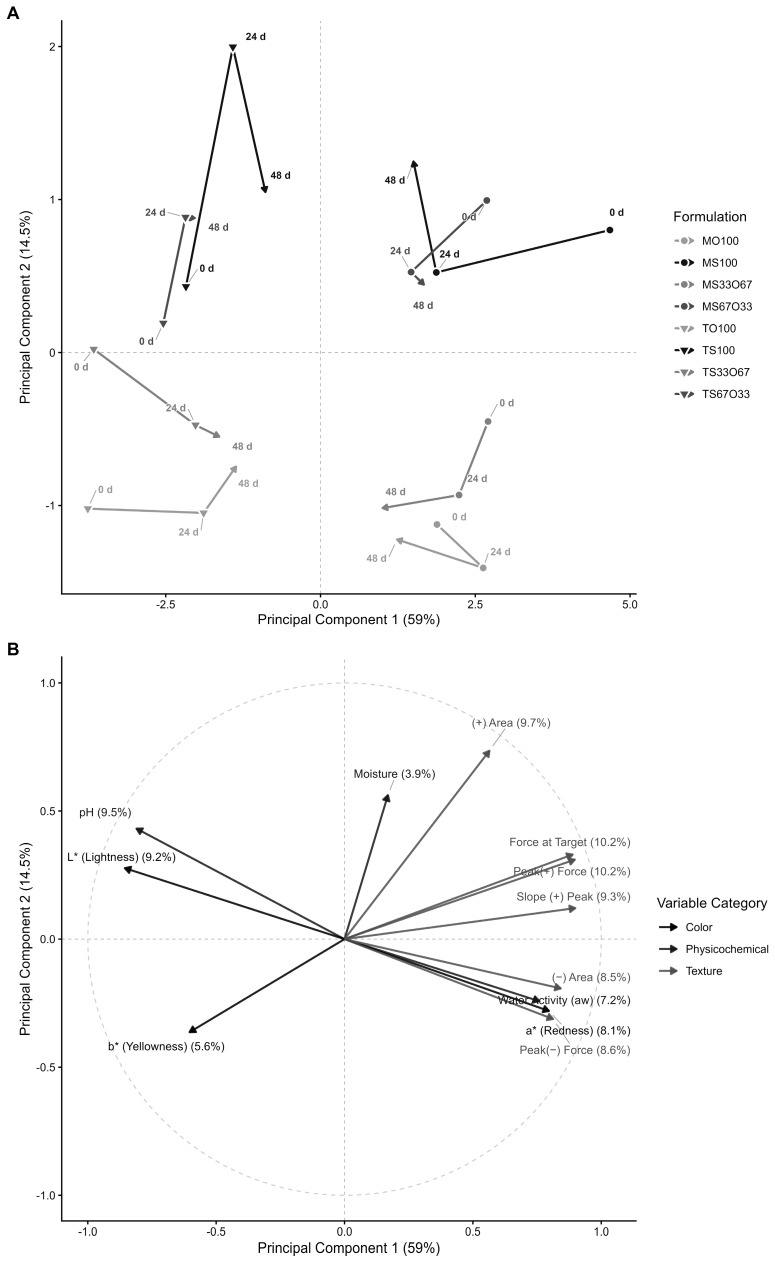
Time-related trajectory plot (**A**) and PCA loading plot (**B**) of cookie quality during storage. Vectors in Panel B indicate variable direction, and are labeled with their parenthetical total contribution percentage across both components (PC1 + PC2). T = Wheat-based; M = Mesquite-based; S = Shortening; O = Oleogel. Storage time points: 0 d (fresh), 24 d, 48 d.

The temporal principal component analysis (PCA2), conducted on measurements taken on Days 0, 24, and 48 of storage, explained 73.5% of the total variance (PC1 = 59.0%; PC2 = 14.5%) (Figure 3).

#### 3.2.2. ANOVA of Storage Effects

**Figure 4:**
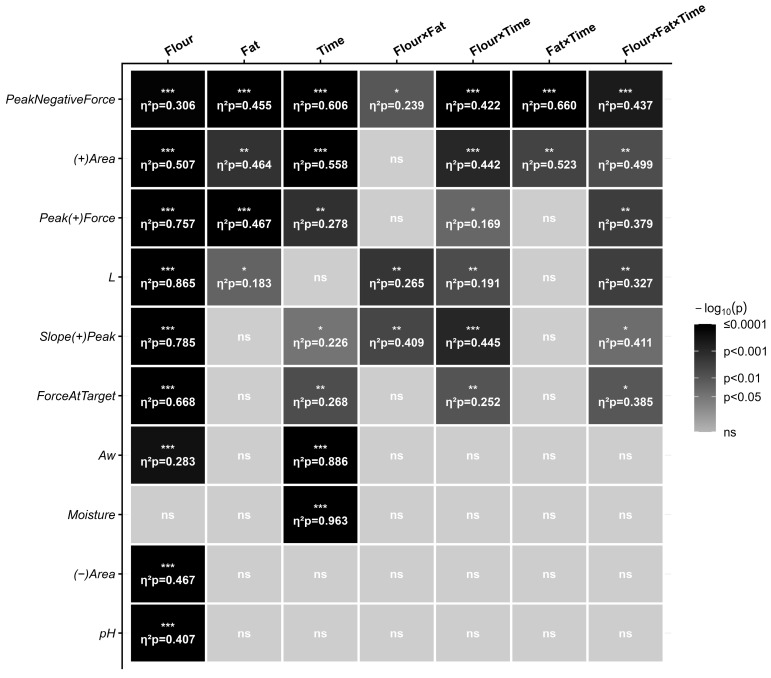
Heatmap of ANOVA effects on storage quality variables (PCA 2) in wheat- and mesquite-based cookies. Cell values display the statistical significance alongside the practical effect size based on partial eta-squared (η^2^p); color intensity represents −log10(*p*-value). Stars = significance level *** *p* < 0.001, ** *p* < 0.01, * *p* < 0.05, ns = not significant.

The ANOVA of storage effects, incorporating Flour Type, Fat Mix, and Storage Time (Days 0, 24, 48) as factors, is presented in Figure 4. Storage time emerged as a highly significant factor across the dataset for moisture-related and specific textural variables. Both moisture content and water activity (a*_w_*) varied significantly with time, which tracks with a continuous water redistribution during storage across all formulation. Cookie Peak Negative Force and (+) Area were significantly associated with storage time, which maps alongside progressive hardening and loss of crispness.

The progressive increase in moisture content from approximately 3.10–3.60% on Day 0 to 13.59–16.80% on Day 48 highlights the hygroscopic nature of the cookie matrix, particularly within the mesquite flour-containing systems (Table 2).

**Figure 5:**
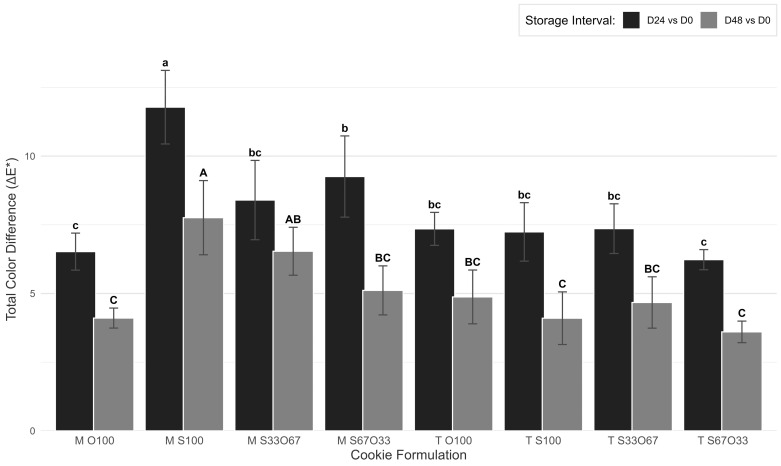
Total color difference (ΔE*) across cookie formulations after 24 and 48 days of storage. Bars represent Mean ± SD (n = 3); different superscript letters indicate significant differences (Tukey HSD, *p* < 0.05; lowercase = D24, uppercase = D48).

Total Δ*E* (Days 24 and 48 vs. Day 0) was consistently higher in the M-series than T-series, which points to lower color stability in the mesquite formulations (Figure 5). MS100 showed the greatest change on Day 24 (11.78 ± 1.34) and remained the most affected on Day 48 (7.76 ± 1.35). In contrast, TS67O33 (3.60 ± 0.39) and TS100 (4.10 ± 0.96) exhibited the lowest Δ*E* on Day 48.

Considering the high lipid content of the developed cookies, the evaluation of oxidative stability through peroxide value (PV), TBARS, Rancimat, and related analyses is of considerable importance. These studies are currently in progress and are expected to provide further insight into the chemical stability and shelf-life potential of the oleogel-based formulations.

### 3.3. Sensory Evaluation

**Figure 6:**
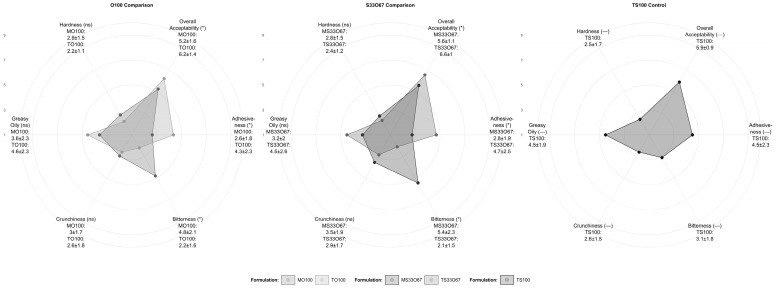
Radar chart corresponding to the quantitative descriptive analysis (QDA) of cookies, evaluated based on five sensory attributes. The intensities were rated by trained panelists using a 9-point scale, where 1 represents low intensity and 9 represents high intensity. Values = Mean ± SD (n = 3). Intra-panel comparisons between formulations: * = significant (*p* < 0.05); ns = not significant.

Sensory evaluation showed clear differences driven by mesquite flour and fat type across five attributes: greasy/oily perception, bitterness, hardness, crunchiness, and adhesiveness (Figure 6). Bitterness was the most discriminating attribute, with mesquite formulations (MO100, MS33O67) scoring markedly higher (~4.8–5.0) than wheat-based samples (1.8–2.8). Intra-panel analysis confirmed these sharp differences while also identifying significant variations in adhesiveness and acceptability. In contrast, fat type and mesquite flour incorporation did not significantly impact greasy/oily perception, hardness, or crunchiness. Ultimately, bitterness showed a strong inverse correlation with acceptability, serving as the primary driver of reduced consumer preference.

## 4. Discussion

The comparable magnitude of both components (Figure 1) indicates that the variability inherent to the cookie system is distributed across two largely independent axes, reflecting a multi-factorial system in which dough structure and baking dynamics con-tribute almost equally to overall product differentiation. This structural independence suggests that enhancements in mechanical strength do not necessarily correspond to changes in moisture retention or baking performance, reinforcing the need for multivariate frameworks when evaluating reduced-fat or reformulated baked systems [14].

Principal Component 1 (PCA1), a dough structure and lipid mobility axis, was pre-dominantly driven by Cookie Oil Migration (14.2%) and dough strength variables like Positive Area (12.2%), Peak Positive Force (11.8%), and Slope Positive Peak (9.6%). High positive PCA1 scores indicated greater dough mechanical resistance and lower oil migration; negative scores indicated lower structural resistance and higher moisture retention. A counterintuitive finding from the PCA1 biplot was the opposite direction of the oil migration vector relative to dough strength variables, indicating that weaker doughs showed greater oil migration. This may reflect flexible, less adaptable matrices that, under thermal stress during baking, promote phase separation and lipid exudation through microstructural discontinuities [15].

The results for the shortening-based formulations (MS100 and MS67O33) reflecting the known ability of crystalline fats to form cohesive dough networks [16]. In contrast, MO100 fell on the negative PCA1 side (reduced firmness, higher moisture retention, higher oil migration), consistent with reports that oleogels decrease dough viscoelasticity and cohesion while increasing extensibility [17]. Conversely, blended systems such as TS33O67, despite their lower mechanical resistance, demonstrated improved oil immobilization, consistent with the ability of oleogels to retain liquid oil within a three-dimensional network [18]. This finding highlights a critical formulation challenge: mechanical robustness and lipid stability are not inherently aligned and must be balanced strategically.

Principal Component 2 (PCA2), a moisture-thermal transformation axis, was principally driven by Cookie Ash (13.8%), Baking Moisture Retention Index (13.4%), Baking Moisture (11.7%), Bake Loss Rate (10.2%), and Dough Negative Area (12.2%). Positive PCA2 scores were associated with higher ash contents and enhanced moisture retention during the baking process. Conversely, negative scores were indicative of increased bake loss and structural dehydration.

Mesquite flour, distinguished by its elevated mineral content (0.93–0.96% ash) and fiber, exhibited reduced bake loss and enhanced moisture retention when compared to wheat-based systems. This observation aligns with the high water-holding capacity inherent in hydrocolloid-rich matrices, a property that mitigates evaporation during baking processes and augments structural water entrapment [19,20]. Mesquite contains galactomannans and phenolic compounds, which have been shown to promote cohesive networks that limit water mobility [21,22].

The TAG analysis (PCA1 biplot, Figure 1) exhibited clear stratification along two opposing trajectories driven by the fat system. Higher oleogel incorporation resulted in an enrichment of PUFA-rich TAGs, while shortening-based formulations exhibited a greater prevalence of SFA-rich TAGs. Consequently, UFA-rich TAGs exhibited an upward trend with oleogel content, while SFA-rich TAGs were associated with shortening. Within this framework, the M-series displayed a distinct TAG profile compared to the T-series, thereby reinforcing the combined influence of flour matrix and lipid phase on overall composition. Although variations were generally moderate across formulations, TS100 exhibited a pronounced depletion of PUFA-rich TAGs, reflecting the predominance of hydrogenated shortening in its lipid fraction [23].

The PCA biplot (Figure 1) further corroborated these findings by demonstrating a clear separation between the two objective TAG classes (UFA-rich and SFA-rich). SFA-rich TAGs were associated with increased cookie spread and reduced lipid mobility, while PUFA-rich TAGs were associated with oleogel-containing systems. This finding indicates that lipid composition may serve a multifaceted role in baking, functioning not only as a nutritional parameter but also exhibiting a strong correlation with baking performance. The structural characteristics of TAGs, including chain length and degree of saturation, influence a variety of physical properties, including melting behavior, viscosity, and fat redistribution during baking. These structural characteristics, in turn, affect spread and texture [24].

The PCA2 biplot (Figure 2) revealed opposing trends in triacylglycerol (TAG) groups: PUFA-rich TAGs demonstrated a positive correlation with oleogel content, while SFA-rich TAGs exhibited a correlation with the proportion of shortening.

The interaction between flour and fat was found to be statistically significant (*p* < 0.001) for dough textural variables, indicating that the effect of the fat system on dough mechanical properties was dependent on the type of flour used. This significant interaction means that system behavior emerges from their combined effects, underscoring a critical trade-off: greater dough strength co-varies with reduced oil migration; higher moisture retention maps alongside lower spread; and increased spread is associated with decreased lipid mobility and variations in TAG composition [25,26]. Consequently, the optimization of a specific quality attribute invariably entails compromises in other attributes, necessitating a multi-objective formulation strategy.

The Spread Factor and Dough Negative Area exhibited a significant response to the simultaneous influence of all three factors, thereby identifying them as the most sensitive indicators of formulation changes. The analysis revealed that flour type was the exclusive significant driver (*p* < 0.001) for moisture retention index, ash content, and bake loss rate. Conversely, fat mix dominated in effects on oil migration (*p* < 0.001) and was the primary driver of textural parameters (dough positive area, slope (+) peak, peak (+) force).

Wheat-based formulations demonstrated higher spread, attributable to diminished water-binding capacities and augmented free fat availability, thereby facilitating lateral expansion during the baking process [27]. In contrast, mesquite flour exhibited a reduced spread capacity, attributable to its enhanced water absorption and matrix densification properties. The observed variability across oleogel-containing systems corroborates prior findings, which suggest that spread is predominantly influenced by the interplay between lipid structure, viscosity, and the protein-fiber matrix rather than by a solitary compositional factor [28].

The extent of oil migration exhibited significant variation: The TO100 and MO100 samples demonstrated the highest values, with 32.30 and 21.80 milligrams of oil per cookie, respectively. In contrast, the MS100 and TS100 samples exhibited the greatest lipid stability, with 2.10 milligrams of oil per cookie. While oleogel systems generally exhibited higher oil migration due to weaker gel networks [29], the mesquite matrix appeared to mitigate this effect relative to wheat, likely through matrix-mediated entrapment and protein-lipid interactions [19,30]. Ash content revealed a clear compositional dichotomy between the M series (0.93–0.96%) and the T series (0.33–0.41%), reflecting the intrinsically higher mineral content of mesquite flour and its nutritional relevance [31]. The moisture retention levels across all formulations were found to be minimal, with values below 1%, MS33O67 exhibiting the highest value of 0.66%, and the TS series displaying the lowest value of approximately 0.53%.

The high proportion of variance (Figure 3A,B) captured by PC1 reflects a strong and consistent formulation identity signal that persisted throughout the storage period. PC1 was predominantly driven by cookie textural variables (Slope Positive Peak: 11.6%; Force at Target: 11.3%; Peak Positive Force: 11.1%) and color lightness L* (10.6%), effectively and persistently separating mesquite-based (M) from wheat-based (T) formulations. This persistent separation indicates that flour composition acts as a physicochemical anchor, conferring a distinct structural and chromatic signature [20,30]. The variance captured by PC1 demonstrates that flour composition functions as a physicochemical anchor, thereby ensuring the maintenance of formulation identity irrespective of fat type or storage duration.

PC2, characterized by its preponderance of Positive Area (31%), Moisture (18.1%), and pH (10.7%), encapsulated the temporal progression of the system, predominantly linked to moisture redistribution and structural relaxation phenomena (Figure 3A). Furthermore, the trajectory analysis (Figure 3A) revealed pronounced differences in stability among formulations over time. MO100 and MS33O67 demonstrated the shortest and most compact trajectories, suggesting minimal physiochemical alteration over the 48-day period. In contrast, TS100 and MS100 exhibited the longest trajectories. TS100 exhibited a pronounced upward shift along PC2 between Days 0 and 24, indicating substantial moisture accumulation or structural rearrangement, which is consistent with starch retrogradation [32]. In contrast, MS100 demonstrated a progressive shift toward a more moisture-sensitive state as storage progressed. MS67O33 exhibited an erratic V-shaped trajectory, moving leftward at Day 24 before reverting rightward by Day 48. This suggests a two-stage aging mechanism characterized by initial moisture uptake and structural relaxation followed by secondary hardening or lipid redistribution. This behavior is consistent with the complex interplay between partially structured lipid networks and the complex mesquite matrix [33].

The stability biplot (Figure 3B) confirmed the presence of directional clustering. The M-series, located on the right, demonstrates higher force and higher redness (a). In contrast, the T-series, positioned on the left, exhibits higher L and higher pH. Full-shortening formulations (S100) within each group exhibited larger confidence ellipses, indicating greater intra-group variability during storage due to fat recrystallization and polymorphic transitions. Oleogel-rich systems have been shown to exhibit shorter and more directionally stable trajectories in comparison to their full-shortening counterparts. This phenomenon indicates an augmented resistance to storage-induced transformations.

The flour type remained the dominant main effect (Figure 4) (*p* < 0.001 for nearly all variables except moisture content). The observed dominance is indicative of the pivotal role that flour plays as a structural anchor. The proteins, starches, and fibers present in flour establish the matrix, thereby dictating water distribution, network development, and Maillard-driven color formation. This principle remains consistent irrespective of the fat type or the duration of storage. The Flour × Time interaction was found to be statistically significant for Lightness L* and Force at Target, thereby confirming the hypothesis that color and structural aging rates vary with flour composition. The effects of fat and fat × time on peak negative force and positive area were found to be statistically significant. This phenomenon is presumably attributable to the fat’s substantial solid fat content (SFC), wherein elevated levels of saturated fatty acids propel the formation of cohesive, more robust matrices, thereby enhancing dough adhesiveness and cookie breaking strength [34].

A triple interaction (Flour × Fat × Time) was identified as significant for Lightness L* (*p* < 0.01) and Force at Target (*p* < 0.05). This finding underscores that color changes are governed by complex, multi-factor dynamics that cannot be predicted from single-factor effects alone. Furthermore, pH and (-) Area were driven exclusively by Flour Type, showing no significant time, fat, or interaction effects. This confirms them as stable compositional fingerprints for quality control.

Mesquite flour’s Type II sigmoidal sorption isotherms indicate a high degree of atmospheric moisture affinity [10], thereby explaining the greater moisture uptake observed in M-series formulations. These values align with reported levels for mesquite flour-based baked goods [35]. Notwithstanding the augmented bulk moisture content, the water activity remained below the 0.60 safety threshold, stabilizing at 0.21–0.22 by Day 48 [36]. This apparent dissociation is indicative of the strong, macromolecular water-binding capacity established earlier, which immobilizes water and reduces its thermodynamic availability [19,33,37]. The fat type exhibited no substantial impact on moisture or water activity, thereby substantiating the hypothesis that oleogels do not introduce additional water or modify moisture dynamics [38]. Nonetheless, oleogel formulations, particularly MO100, demonstrated reduced moisture accumulation, indicating that the structured lipid network functions as a partial water barrier [39].

The pH profiles exhibited stability throughout the storage period (Table 2), with distinct separation between the wheat-based formulations (6.72–6.94) and the mesquite-based formulations (6.02–6.53). This outcome aligns with the established findings regarding the role of mesquite flour’s acidic phenolic composition, which comprises compounds such as caffeic acid, p-coumaric acid, and flavonoid glycosides [30,31]. The experimental observations reported in the current study are consistent with the previously documented ranges, thereby substantiating the assertion that even partial substitution is sufficient to induce a sustained acidification of the matrix. A slight decrease in pH at Day 24, followed by a partial recovery at Day 48, suggests a buffering capacity of mesquite proteins and polysaccharides [37,40].

Color development was among the most complex attributes evaluated (Table 2), with a significant Flour × Fat × Time interaction for lightness (L*). Mesquite-based formulations exhibited lower L* and higher a* values compared to wheat-based cookies over the duration of the storage period. This observation is indicative of the presence of natural pigments (tannins, anthocyanins), as well as enhanced Maillard reactivity derived from mesquite flour’s reducing sugars and amino acids [30].

All formulations exhibited a decrease in the color difference (Δ*E*) from Day 24 to Day 48, indicating a non-linear trend with an initial rapid color change followed by a period of partial stabilization. This phenomenon may be associated with the presence of antioxidant phenolics, such as vicenin II and isoschaftoside, which have been shown to impede oxidation and browning reactions [30,31]. Furthermore, oleogel-based systems (e.g., MO100; Δ*E*(D24) = 6.52 vs. MS100 = 11.78) exhibited reduced color variation, thereby substantiating the function of structured lipid networks in diminishing oxygen diffusion, pigment oxidation, and overall color instability [39].

Textural alterations manifested in a biphasic manner during the 48-day storage interval, exhibiting an initial hardening phase (Days 0–24) and a subsequent stabilization or slight softening phase by Day 48. This phenomenon is predominantly ascribed to starch retrogradation, specifically amylopectin recrystallization, which engenders increased rigidity during the initial storage phase [41]. Moisture redistribution is another factor that contributes to this phenomenon. Initially, it promotes firming, and subsequently, it leads to partial softening as environmental moisture is absorbed [42]. For instance, the measurement of maximum force (Peak (+)) for MO100 increased from 36.81 Newtons (Newtons) on Day 0 to 46.74 Newtons on Day 24, then decreased to 34.86 Newtons on Day 48. MS100 exhibited the highest degree of hardness throughout the experiment (54.41 N at Day 48), suggesting enhanced structural integrity. Water activity exhibited an increase across all formulations, from 0.11 to 0.15 to 0.21–0.22 by Day 48, thereby confirming moisture ingress and providing support for the observed softening after Day 24.

Mesquite-based formulations consistently exhibited greater hardness and crispness compared to wheat-based samples, as evidenced by higher Slope (+) Peak values and a more brittle fracture behavior, which agrees with previous sensory reports [43]. This phenomenon is attributed to the dense matrix of starch and the absence of a continuous gluten network, as well as distinct protein–starch interactions [30,44].

The fat type exhibited a substantial influence on the textural alterations that occurred over time (Fat × Time interaction, Peak Negative Force). Oleogel-containing formulations demonstrated reduced hardening rates and enhanced stability, likely attributable to diminished water mobility and mitigated starch retro-gradation [45]. Their lower hardness is consistent with weaker structuring and reduced aeration capacity compared to plastic fats [39]. In mesquite systems, this softening effect was less pronounced, suggesting that interactions between oleogels and the alternative flour matrix partially compensate for reduced lipid structuring. This phenomenon may explain the enhanced stability observed in formulations such as MO100 and MS33O67, where the components exhibit a synergistic effect [20]. Furthermore, mesquite cookies exhibited reduced adhesiveness, resulting in a less sticky and more friable structure [30,46].

Greasy/oily and adhesive properties were found to be moderate across all samples, with a score of less than five on a scale of one to five. The formulations containing exclusively wheat flour (TS100, TS33O67, TO100) exhibited a propensity for higher scores (approximately 4.4–4.5 on greasy scale; >4.3 on adhesiveness scale), suggesting a texture that is moister and adhesive. In contrast, mesquite-based samples exhibited lower values (~3.2–3.6 on greasy scale; ~2.0 on adhesiveness scale), indicative of a drier, less cohesive structure due to the highly absorptive flour matrix. This structure limits free moisture and lipid interactions, resulting in a product that is less oily and less sticky [30,46].

The hardness and crunchiness of the samples remained at a low level (2.1–3.3), with no significant differences observed, indicating that neither the substitution of flour nor the incorporation of oleogel significantly altered the mechanical texture of the samples.

The extent of global acceptability, measured using a 9-point scale, exhibited modest variability across the various formulations. The highest scores were observed for TS33O67 (6.56, “like moderately”), followed by TO100 (6.21) and TS100 (5.89). These results suggest that the low bitterness and familiar flavor profiles present in wheat-based samples are advantageous. Lower scores were recorded for MS33O67 (5.58) and MO100 (5.25), which were within the neutral range. This was primarily due to higher bitterness levels. The variability of MO100 was the greatest (SD = 1.59), indicating a polarizing response to the mesquite flavor among the assessors. In contrast, TS100 exhibited the lowest variability (SD = 0.94), suggesting consistent acceptance. In general, the level of acceptability of mesquite flour was marginally diminished due to the presence of bitterness, although the impact was only moderate. To minimize bitterness, it is essential to optimize flavor balance through the use of high-sugar matrices or the addition of masking agents, such as vanilla, chocolate, or cinnamon, to suppress bitter notes while maintaining functionality.

Although minor variations in acceptability were observed, these were primarily dictated by the flour matrix rather than the fat system. This finding suggests that oleogels have considerable potential as a functional shortening substitute, with minimal impact on overall sensory characteristics.

## 5. Conclusions

The combined findings demonstrate that the quality of mesquite- and wheat-based cookie systems is governed by a dual-mechanism framework. In this framework, dough mechanical strength and lipid physical stability operate as partially independent forces, while moisture dynamics constitute a formulation-modulated factor. In the context of the experimental conditions established for the present study, oleogel substitution emerges as a promising formulation approach for enhancing the nutritional quality of cookies. This approach involves a reduction in saturated fats without compromising the physical integrity of the product over the course of a 48-day storage period. In addition, while oleogels effectively replicated the mouthfeel of conventional fats, the incorporation of mesquite flour resulted in discernible sensory disparities, particularly regarding bitterness, which had a deleterious effect on overall acceptability. In terms of practical implementation, formulations employing a 67% oleogel substitution in wheat-based systems (TS33O67) are advised, as they have demonstrated optimal consumer acceptability. Further optimization is required for mesquite flour-enriched products; future research should explore strategies for reducing bitterness to enhance the commercial viability of these cookie products with improved nutritional properties and study their chemical stability.

## Figures and Tables

**Table 1:** Dough rheology and baked product properties of wheat- and mesquite-based cookies formulated with shortening or oleogel.

	Dough (+) Area (N·s)	Dough (−) Area (N·s)	Moisture Retention (%)	Spread Factor	Oil Migration (mg oil/Cookie)	Ash (%)
MO100	6.25 ± 0.37 ^d^	−1.09 ± 0.02 ^c^	0.63 ± 0.01 ^ab^	1.56 ± 0.15 ^d^	21.80 ± 2.50 ^a^	0.94 ± 0.08 ^a^
MS100	16.71 ± 2.08 ^a^	−0.60 ± 0.23 ^b^	0.596 ± 0.004 ^bc^	1.83 ± 0.11 ^bcd^	2.10 ± 0.20 ^d^	0.93 ± 0.03 ^a^
MS33O67	9.85 ± 0.46 ^c^	−0.16 ± 0.11 ^a^	0.66 ± 0.02 ^a^	1.92 ± 0.11 ^bc^	8.00 ± 0.30 ^b^	0.96 ± 0.03 ^a^
MS67O33	13.54 ± 0.82 ^b^	−0.14 ± 0.04 ^a^	0.58 ± 0.03 ^bcd^	1.99 ± 0.18 ^b^	4.70 ± 0.30 ^c^	0.95 ± 0.01 ^a^
TO100	3.81 ± 0.34 ^e^	−1.11 ± 0.01 ^c^	0.545 ± 0.003 ^cde^	1.65 ± 0.11 ^cd^	32.30 ± 5.13 ^a^	0.33 ± 0.02 ^b^
TS100	14.74 ± 1.11 ^ab^	−1.93 ± 0.02 ^d^	0.527 ± 0.004 ^e^	2.46 ± 0.10 ^a^	2.10 ± 0.53 ^d^	0.41 ± 0.10 ^b^
TS33O67	5.72 ± 0.32 ^d^	−1.09 ± 0.08 ^c^	0.539 ± 0.001 ^de^	2.48 ± 0.08 ^a^	11.33 ± 2.85 ^b^	0.40 ± 0.08 ^b^
TS67O33	8.62 ± 0.42 ^c^	−1.93 ± 0.01 ^d^	0.530 ± 0.001 ^e^	2.33 ± 0.18 ^a^	8.17 ± 1.10 ^b^	0.37 ± 0.04 ^b^

Mean ± SD (n = 3). Different superscript letters within the same column indicate significant differences (Tukey HSD, *p* < 0.05). T = wheat-based; M = mesquite-based; S = shortening; O = oleogel.

**Table 2:** Storage quality variables (24 and 48 days) for wheat- and mesquite-based cookies with shortening or oleogel.

	Slope (+) Peak (N/s)			Peak (+) Force (N)			Peak (−) Force (N)			(+) Area (N·s)			Water Activity (*a_w_*)			pH			Lightness (L*)		
	0 d	24 d	48 d	0 d	24 d	48 d	0 d	24 d	48 d	0 d	24 d	48 d	0 d	24 d	48 d	0 d	24 d	48 d	0 d	24 d	48 d
MO100	18.32 ± 1.54 ^b^	16.95 ± 2.58 ^abc^	19.65 ± 0.83 ^bc^	36.81 ± 2.34 ^b^	46.74 ± 4.56 ^a^	34.86 ± 1.76 ^cd^	−0.14 ± 0.02 ^ab^	−0.14 ± 0.01 ^ab^	−0.136 ± 0.003 ^a^	80.25 ± 1.02 ^cd^	89.49 ± 7.38 ^bc^	86.83 ± 0.98 ^bc^	0.14 ± 0.01 ^a^	0.21 ± 0.01 ns	0.223 ± 0.001 ns	6.48 ± 0.25 ^cd^	6.03 ± 0.05 ^c^	6.30 ± 0.11 ^c^	69.85 ± 1.49 ^b^	70.37 ± 1.88 ^b^	67.21 ± 0.62 ^c^
MS100	33.13 ± 4.31 ^a^	22.61 ± 1.97 ^ab^	25.56 ± 0.96 ^ab^	50.93 ± 1.80 ^a^	53.06 ± 0.66 ^a^	54.41 ± 2.48 ^a^	−0.125 ± 0.002 ^a^	−0.15 ± 0.01 ^ab^	−0.19 ± 0.01 ^abc^	139.51 ± 16.48 ^a^	137.81 ± 6.06 ^a^	145.57 ± 8.60 ^a^	0.143 ± 0.003 ^a^	0.206 ± 0.004	0.220 ± 0.004	6.38 ± 0.16 ^d^	6.34 ± 0.06 ^bc^	6.49 ± 0.04 ^abc^	66.62 ± 1.44 ^b^	74.31 ± 1.12 ^b^	71.79 ± 0.86 bc
MS33O67	22.23 ± 5.30 ^b^	23.08 ± 2.94 ^a^	17.19 ± 4.16 ^cd^	36.29 ± 3.32 ^b^	45.79 ± 2.05 ^ab^	38.76 ± 4.10 ^bc^	−0.13 ± 0.01 ^a^	−0.12 ± 0.01 ^a^	−0.13 ± 0.01 ^a^	97.17 ± 9.85 ^bc^	102.45 ± 2.30 ^b^	99.81 ± 0.30 ^bc^	0.15 ± 0.01 ^a^	0.21 ± 0.01	0.222 ± 0.004	6.44 ± 0.16 ^d^	6.04 ± 0.02 ^c^	6.42 ± 0.06 ^bc^	67.17 ± 2.31 ^b^	71.42 ± 0.76 ^b^	72.79 ± 0.24 ^b^
MS67O33	23.52 ± 1.53 ^b^	24.94 ± 2.70 ^a^	28.89 ± 3.75 ^a^	46.74 ± 3.63 ^a^	53.54 ± 6.24 ^a^	45.75 ± 1.29 ^ab^	−0.13 ± 0.01 ^a^	−0.18 ± 0.01 ^ab^	−0.16 ± 0.02 ^ab^	121.39 ± 8.95 ^ab^	140.66 ± 0.02 ^a^	109.19 ± 1.39 ^b^	0.14 ± 0.01 ^a^	0.203 ± 0.004	0.220 ± 0.002	6.53 ± 0.25 ^bcd^	6.28 ± 0.04 ^c^	6.50 ± 0.02 ^abc^	69.09 ± 1.92 ^b^	72.41 ± 1.78 ^b^	68.84 ± 1.73 ^bc^
TO100	8.94 ± 0.93 ^c^	11.77 ± 0.42 ^c^	9.42 ± 3.26 ^de^	19.58 ± 1.89 ^d^	21.36 ± 0.75 ^c^	23.13 ± 3.78 ^e^	−0.24 ± 0.03 ^d^	−0.20 ± 0.02 ^bc^	−0.220 ± 0.001 ^bc^	69.67 ± 9.71 ^d^	74.95 ± 5.38 ^c^	90.21 ± 11.30 ^bc^	0.117 ± 0.003 ^b^	0.198 ± 0.001	0.219 ± 0.001	6.82 ± 0.20 ^abc^	6.80 ± 0.04 ^a^	6.82 ± 0.03 ^ab^	83.62 ± 2.03 ^a^	86.41 ± 1.13 ^a^	86.16 ± 0.49 ^a^
TS100	9.94 ± 2.53 ^c^	14.68 ± 2.24 ^bc^	8.38 ± 0.02 ^e^	29.15 ± 3.15 ^c^	37.31 ± 2.85 ^b^	27.52 ± 0.64 ^de^	−0.18 ± 0.02 ^bc^	−0.313 ± 0.002 ^d^	−0.22 ± 0.02 ^bc^	102.58 ± 14.78 ^bc^	141.04 ± 8.59 ^a^	109.66 ± 7.95 ^b^	0.12 ± 0.01 ^b^	0.196 ± 0.005	0.222 ± 0.002	6.90 ± 0.03 ^a^	6.90 ± 0.01 ^a^	6.88 ± 0.02 ^a^	86.55 ± 2.67 ^a^	88.80 ± 1.12 ^a^	87.14 ± 1.84 ^a^
TS33O67	9.02 ± 0.76 ^c^	11.16 ± 1.05 ^c^	8.20 ± 2.57 ^e^	20.40 ± 2.49 ^d^	22.11 ± 1.55 ^c^	24.69 ± 0.02 ^e^	−0.21 ± 0.03 ^cd^	−0.18 ± 0.02 ^abc^	−0.19 ± 0.01 ^abc^	83.20 ± 0.55 ^cd^	92.72 ± 2.80 ^bc^	76.17 ± 5.82 ^c^	0.12 ± 0.01 ^b^	0.19 ± 0.01	0.214 ± 0.002	6.94 ± 0.21 ^a^	6.73 ± 0.06 ^ab^	6.71 ± 0.02 ^abc^	86.21 ± 2.33 ^a^	87.91 ± 0.80 ^a^	86.93 ± 0.32 ^a^
TS67O33	8.86 ± 0.96 ^c^	9.56 ± 1.08 ^c^	8.89 ± 1.73 ^e^	26.81 ± 1.98 ^c^	28.00 ± 1.80 ^c^	26.39 ± 3.79 ^de^	−0.17 ± 0.03 ^abc^	−0.24 ± 0.04 ^c^	−0.23 ± 0.01 ^c^	91.20 ± 1.86 ^cd^	109.69 ± 3.80 ^b^	95.57 ± 6.02 ^bc^	0.12 ± 0.01 ^b^	0.19 ± 0.01	0.216 ± 0.001	6.84 ± 0.12 ^ab^	6.84 ± 0.06 ^a^	6.93 ± 0.06 ^a^	87.01 ± 0.74 ^a^	88.32 ± 0.61 ^a^	88.50 ± 0.22 ^a^

Mean ± SD (n = 3). Different superscript letters within the same column indicate significant differences (Tukey HSD, *p* < 0.05); ns indicates no significant difference. Shading (Dunnett, 24d and 48d vs. 0d): darker = stronger significance (white: *p* > 0.05, gray: ≈0.05, dark gray: <0.01, black: ≤0.001). Day 0 (unshaded) is the baseline. T = wheat-based; M = mesquite-based; S = shortening; O = oleogel.

## Data Availability

The original contributions presented in this study are included in the article. Further inquiries can be directed to the corresponding author.
